# Safety of surgical hip dislocation in femoral head fracture and dislocation (FHFD) and avascular necrosis risk factor analysis of FHFD: midterm results confirmed by SPECT/CT and MRI

**DOI:** 10.1186/s13018-022-03160-y

**Published:** 2022-05-16

**Authors:** Yong-Cheol Yoon, Chang-Wug Oh, Joon-Woo Kim, Jeong Heo, Hyung Keun Song

**Affiliations:** 1 Orthopedic Trauma Division, Trauma Center, Gachon University College of Medicine, Namdong-gu, Incheon, Republic of Korea; 2 Department of Orthopedic Surgery, School of Medicine, Kyungpook National University, Kyungpook National University Hospital, 130 Dongdeok‑ro, Chung‑gu, Daegu, 41944 Republic of Korea; 3 Department of Orthopedic Surgery, Ajou University School of Medicine, Yeongtong-gu, Suwon-si, Gyeonggi-do Republic of Korea

**Keywords:** Femoral head fracture and dislocation, Trochanteric flip osteotomy, Surgical hip dislocation, Avascular necrosis, Displaced femoral neck fractures

## Abstract

**Background:**

The study aim was to report the treatment outcomes of trochanteric flip osteotomy (TFO) with surgical hip dislocation (SHD) for femoral head fracture and dislocation (FHFD) and to investigate the risk factors for avascular necrosis (AVN) of the femoral head.

**Methods:**

The data of 34 patients (29 men, 5 women; mean age 37.9 years) diagnosed with FHFD and treated with TFO with SHD between May 2009 and February 2018 with an average follow-up period of 5.1 years (range 2.8–10.5 years) were analyzed. Clinical outcomes were evaluated using the Merle d'Aubigné–Postel score and Thompson–Epstein Scale. Radiologic outcomes were classified according to the Matta classification. AVN was confirmed using magnetic resonance imaging or single-photon emission computed tomography/computed tomography. The occurrence of complications was examined, and factors influencing complications, AVN.

**Results:**

Regarding the Pipkin’s classification, there were 7 patients with type II, 2 patients with type III, and 25 patients with type IV fractures. Posterior wall fractures accompanied all associated acetabular fractures in the patients with Pipkin type IV fractures. Radiologically, the union of acetabular and femoral head fractures was observed within 6.1 months on average (range 4–10 months) in 32 patients, except two patients who developed femoral head AVN. Clinically, the average Merle d'Aubigné–Postel score was 14.4 points (range 8–17 points), and 22 patients had good or excellent results on the Thompson–Epstein Scale. Two patients developed femoral head AVN with both having displaced femoral neck fractures associated with FHFD. AVN was significantly correlated with femoral neck fractures (P = 0.000).

**Conclusion:**

TFO with SHD is a safe and useful approach for the treatment of FHFD. Particular attention should be paid when treating femoral head fractures associated with displaced femoral neck fractures because of the high risk of AVN development.

## Background

Femoral head fractures are intra-articular fractures, and restoration of the articular surface through accurate fracture reduction is of paramount importance [1]. The prognosis of these fractures depends on the accuracy of the reduction and stability of fixation. Traumatic hip dislocation accompanied by femoral head fracture is frequently associated with damage to other organs and combined fractures and may cause complications, such as avascular necrosis (AVN) of the femoral head, traumatic arthritis, and heterotopic ossification (HO) [2, 3]. Therefore, prompt and accurate treatment in the early stage of the injury and continuous follow-up are required [4, 5].

Wide visualization of the lesion site is essential for approach, reduction, and plate fixation for the anatomic reduction of femoral head fractures and associated acetabular fractures. Conventionally, the Smith–Petersen anterior, Kocher–Langenbeck posterior, and anteroposterior multidirectional approaches are used for visualization [6, 7]. Using the posterior approach alone provides a limited view of the superoposterior region and superior dome of the acetabulum [8]. However, surgical hip dislocation (SHD) via trochanteric flip osteotomy (TFO) can overcome this limitation and facilitate wide visualization of the operative field, thereby allowing accurate evaluation of the intra-articular lesion and anatomic reduction of fragments of the femoral head and acetabular fractures [9]. Henle et al. performed reduction and fixation using the SHD method in 12 patients with femoral head fractures associated with posterior hip dislocation, and they achieved good or excellent results in 10 (83.3%) patients [10].

To the best of our knowledge, no study has reported relatively long-term follow-up data after SHD, and only case reports have been published because of the low incidence of femoral head fractures. Despite the good outcomes of surgical treatment, no clear treatment guidelines have been proposed yet. Previous studies have confirmed that AVN detected on simple radiographs can develop after SHD performed for traumatic femoral head fracture and dislocation (FHFD) [11]; however, none of the studies performed precise examinations using single-photon emission computed tomography (SPECT)/computed tomography (CT) or magnetic resonance imaging (MRI). Furthermore, no study has described the risk factors for AVN as a serious complication of traumatic hip dislocation associated with femoral head fractures.

Therefore, in this study, patients diagnosed with FHFD and treated with open reduction and internal fixation via TFO with SHD were followed up for a relatively long period of more than 5 years. Their outcomes were analyzed based on clinical and radiologic evaluations, and AVN development was confirmed using MRI or SPECT/CT. Moreover, the factors influencing complications, AVN development, and clinical outcomes were analyzed.

## Materials and methods

### Study population

A total of 42 patients were diagnosed with FHFD, and they underwent TFO with SHD at a level I trauma center between May 2009 and February 2018. After applying the exclusion criteria, 34 patients who underwent MRI or SPECT/CT were followed up for at least 2.5 years and were included in the study, thereby allowing retrospective review of medical records and radiographs. The exclusion criteria were as follows: (1) previous femoral head AVN; (2) physical separation due to open femoral head epiphysis; (3) pre-existing hip joint degenerative osteoarthritis; (4) developmental dysplasia of the hip; (5) joint dysmorphism; and (6) femoral head fracture alone. The 34 patients comprised 29 men and 5 women with a mean age of 37.9 years (range 16–77 years), and the average follow-up period was 5.1 years (range 2.8–10.5 years) (Table 1). The study design and data collection were approved by the institutional review board of the Human Experimental and Ethics Committee of our hospital (approval no. KNUH 2020-12-010).

**Table 1:** Demographic data of patients

Patient #	Age	Sex	Injury mechanism	Injured site	Associated injury	Associated orthopedic fracture	Femur head fracture classification (Pipkin type)	If Pipkin IV, Acetabular fracture classification	Closed reduction time (h)	Reason for failed closed reduction
1	63	M	TA	Rt	MRF with hemothorax	None	II	N/A	6	N/A
2	38	M	TA	Rt	Liver laceration, sciatic nerve palsy	None	II	N/A	6	N/A
3	18	M	TA	Rt	None	None	II	N/A	Irreducible	Femoral head impaction
4	65	M	TA	Rt	MRF, facial bone fx	Rt. Tibia shaft fracture,	II	N/A	4	N/A
5	31	M	TA	Rt	None	Lt. femur shaft fx, Rt. Tibia shaft fx	II	N/A	8	N/A
6	16	M	TA	Lt	None	Lt. femur shaft fx, Lt. tibia shaft fx	II	N/A	18	N/A
7	77	M	TA	Lt	Brain hemorrhage	None	II	N/A	16	N/A
8	44	M	TA	Rt	None	Lt. tibia open fracture	III	N/A	Irreducible	Associated neck fracture
9	31	M	TA	Lt	None	Lt. patella fx	III	N/A	Irreducible	Associated neck fracture
10	43	M	TA	Rt	MRF, hemopneumothorax	None	IV	Posterior wall	7	N/A
11	36	M	TA	Rt	None	Both tibia shaft fx	IV	Posterior wall	2	N/A
12	28	M	Fall down	Rt	None	None	IV	Posterior wall	1	N/A
13	21	M	TA	Rt	Liver laceration	None	IV	Posterior wall	Failed	Two times trial but failed
14	36	M	TA	Lt	None	Lt. scapula fx	IV	Posterior wall	6	N/A
15	29	M	TA	Rt	MRF, liver laceration, sciatic nerve palsy	None	IV	Posterior wall	7	N/A
16	20	M	Fall down	Rt	None	None	IV	Posterior wall	4	N/A
17	39	F	TA	Lt	None	None	IV	Posterior wall	12	N/A
18	28	M	TA	Lt	Scrotal laceration	Rt. 5th Metacarpal fx	IV	Posterior wall	6	N/A
19	34	M	TA	Lt	None	Rt. Distal femur fx	IV	Posterior wall	4	N/A
20	30	M	TA	Rt	Aortic dissection	None	IV	Posterior wall	7	N/A
21	34	M	TA	Rt	Brain hemorrhage	Rt. Patella fx	IV	Posterior wall	3	N/A
22	70	F	TA	Rt	Sciatic nerve palsy	Rt femur shaft fx Degloving injury Lt pelvic bone fx	IV	Posterior wall	5	N/A
23	59	M	TA	Lt	Small bowel injury	None	IV	Posterior wall	4	N/A
24	32	F	TA	Lt	None	None	IV	Posterior wall	10	N/A
25	32	M	TA	Rt	Brain hemorrhage	None	IV	Posterior wall	6	N/A
26	42	F	TA	Rt	None	Rt. Tibia PCL avulsion fx	IV	Posterior wall	13	N/A
27	24	M	TA	Rt	Sciatic nerve palsy	None	IV	Posterior wall	5	N/A
28	63	M	TA	Lt	Sternum fx, MRF	Lt. tibia shaft fx,	IV	Posterior wall	3	N/A
29	31	M	TA	Lt	None	None	IV	Posterior wall	6	N/A
30	17	M	TA	Rt	Facial bone fx	None	IV	Posterior wall	failed	Two times trial but failed
31	54	F	TA	Lt	None	Lt. both forearm fx	IV	Posterior wall	12	N/A
32	26	M	TA	Lt	Liver laceration	both femur shaft fx Rt femur neck fx	IV	posterior wall	7	N/A
33	31	M	TA	Rt	Brain hemorrhage	None	IV	Posterior wall	3	N/A
34	47	F	TA	Rt	None	None	IV	Posterior wall	Irreducible	Associated neck fracture

M, male; F, female; TA, traffic accident; Rt., right; Lt., left; MRF, multiple rib fracture; N/A, not applicable; fx., fracture; PCL: posterior cruciate ligament

### Surgical technique

The patients were placed under general anesthesia in the lateral decubitus position, and the affected area was visualized. Using the modified Gibson posterior approach, a straight long-skin incision was made along the long axis of the femoral shaft that passed through the greater trochanter (GT) [12]. Without dissecting the gluteus maximus, the area between the tensor fasciae latae and anterior border of the gluteus maximus was dissected. Efforts to reduce the tension of the sciatic nerve were made through hip extension and knee flexion of the affected leg, and care was taken while handling the soft tissues during the entire procedure to reduce the risk of HO.

**Fig. 1:**
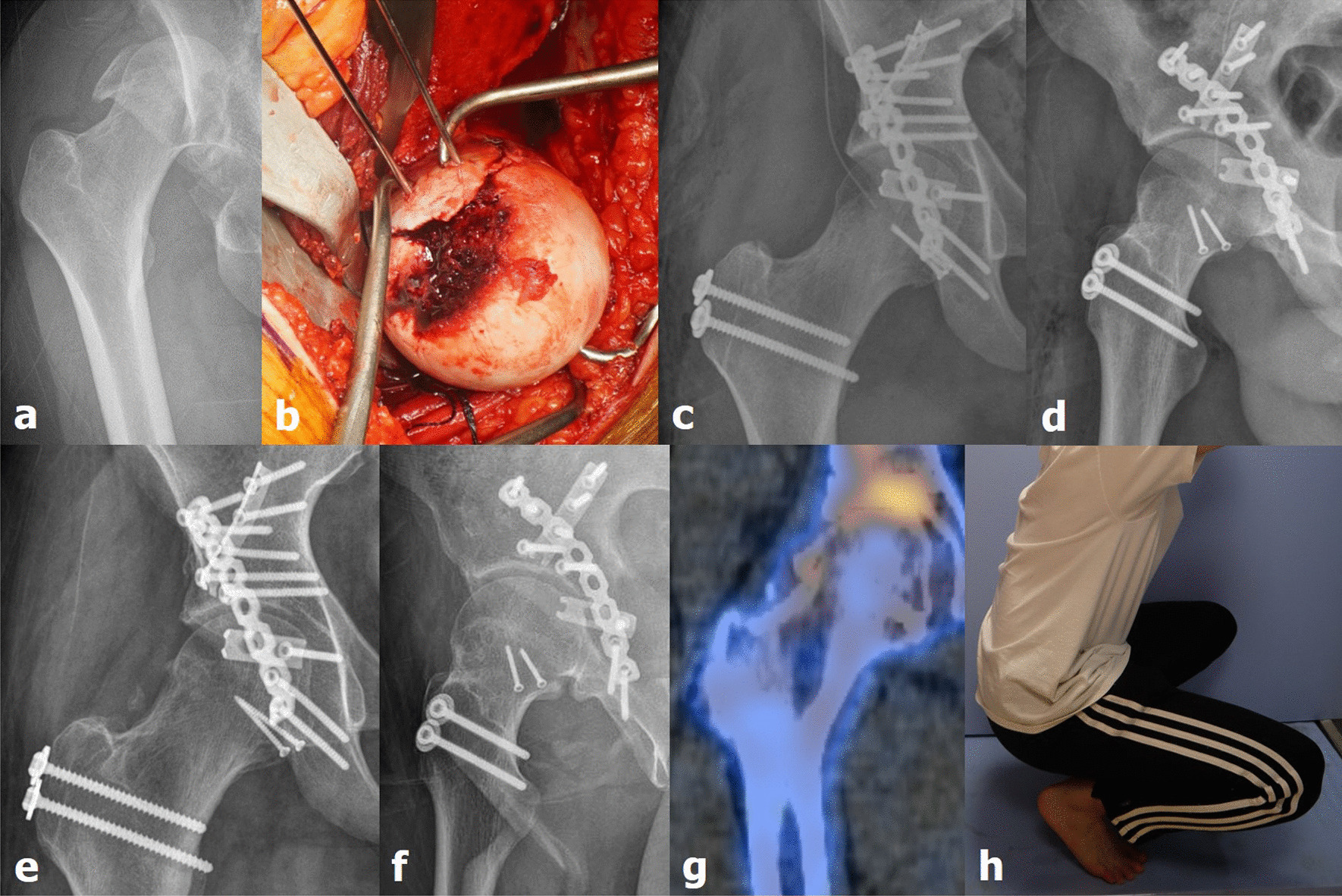
(**a**) Pipkin type IV femoral head fracture and dislocation of the right hip joint owing to an in-car traffic accident (case 14, a 36-year-old man). (**b**) Surgical hip dislocation was performed using the modified Gibson approach. The femoral head fracture was reduced using pointed reduction forceps; fixation was performed using 2.7-mm cortical screws. (**c**, **d**) Stable fixation was achieved, and good congruence of the femoral head was found to be maintained on postoperative radiography. (**e**, **f**) Bone union was achieved 8 months after surgery, and (**g**, **h**) blood supply to the femoral head was found to be well maintained on single-photon emission computed tomography/computed tomography, and the patient fully recovered range of motion

After identifying the gluteus medius and posterior border of the vastus lateralis, TFO was performed. In brief, femoral head fragments were exposed by performing a Z-shaped capsular incision and dislocating the hip joint by gentle external rotation and flexion of the lower extremities. Thereafter, the femoral head fragments were anatomically reduced as much as possible using pointed reduction forceps. The femoral head was fixed with cortical screws or headless compression screws, and the hip joint was reduced by internally rotating the lower extremities. Acetabular fixation using a metal plate or screws was performed in patients with acetabular fractures that required fixation (Fig. 1). Conservative treatment was performed in patients in whom an acetabular fracture was observed, but with small fragments, such that the acetabular fracture did not affect hip joint stability.

### Radiologic and clinical evaluations

Femoral head fractures at the time of injury were categorized using the Pipkin classification, and the time interval from injury to closed reduction of the femoral head was calculated [13]. The reason for fractures was determined when closed reduction failed or was not performed. Associated injuries, fractures, and injury severity scores were also investigated [14]. The fixatives used to fix the femoral head and reduce associated acetabular fractures during surgery were identified. Clinical outcomes were graded as excellent, good, fair, or poor based on the Merle d'Aubigné–Postel score and Thompson–Epstein Scale [15, 16]. Radiologic outcomes were analyzed using the Matta classification by evaluating the union of the fracture and osteotomy sites [17]. The development of AVN of the femoral head (confirmed using MRI or SPECT/CT 1 year after surgery) [18], development of complications, such as HO and traumatic osteoarthritis, and need for additional surgery because of complications were also analyzed [19].

### Statistical analysis

The correlations of complications, femoral head AVN, and functional score with the other factors listed in Tables 1 and 2 were statistically analyzed. In the analysis of factors influencing the development of complications, the chi-square test or Fisher’s exact test was performed for nominal variables and the Kruskal–Wallis test or Mann–Whitney U test for continuous variables. Multivariate categorical regression analysis was performed to analyze the correlation between femoral head AVN and other factors, and multivariate linear regression analysis was performed to analyze the correlation between the functional scores and other factors. According to the Pipkin classification, patients with femoral neck fractures are classified as type III and patients with acetabular fractures are classified as type IV. Some patients had simultaneous femoral neck and acetabular fractures. Hence, the Pipkin type was excluded from the analysis of multicollinearity, and the analysis was performed with femoral neck and acetabular fractures as items [20]. Statistical analysis was performed using SPSS for Windows (version 16.0; SPSS, Chicago, IL, USA), and a *p* value of < 0.05 was considered to indicate statistical significance.

**Table 2:** Treatment results of femoral head fracture and dislocation cases

Patient #	Head fixation method	Acetabular fixation method	Osteotomy site fixation method	AVN evaluation method (MRI/SPECT/CT)	F/u period (Months)	Bone union (months)	Radiologic results (the Matta's criteria)	Functional results (Merle d 'Aubigne score/ Thompson-Epstein scoring scale)	Heterotrophic ossification (Brooker classification)	Complication	Additional surgery
1	2 cortical screws	N/A	2 cortical screws with washer	MRI	58	6	Excellent	15 / Good	Grade 1	GT nonunion	GT nonunion revision surgery
2	3 cortical screws	N/A	2 cortical screws with washer	MRI	38	7	Excellent	16/Excellent	N/A	N/A	N/A
3	2 headless screw	N/A	2 cortical screws	SPECT/CT	63	5	Good	14/Good	N/A	N/A	N/A
4	3 cortical screws	N/A	2 cortical screws with washer	SPECT/CT	34	7	Good	13/Fair	Grade 1	Irritation for GT osteotomy site	GT screw removal
5	3 cortical screws	N/A	2 cortical screws	SPECT/CT	78	4	Good	15/Good	N/A	N/A	N/A
6	3 cortical screws	N/A	2 cortical screws with washer	SPECT/CT	37	5	Excellent	13/Fair	N/A	N/A	N/A
7	3 cortical screws	N/A	2 cortical screws with washer	SPECT/CT	45	7	Excellent	15/Excellent	N/A	N/A	N/A
8	3 cortical screws	N/A	2 cortical screws with washer	SPECT/CT	75	None	Poor	9/Poor	Grade 2	AVN	THRA
9	3 cortical screws	N/A	2 cortical screws with washer	SPECT/CT	38	4	Good	15/Good	N/A	N/A	N/A
10	2 headless screws	Compression plate	2 cortical screws	SPECT/CT	43	7	Good	16/Excellent	N/A	N/A	N/A
11	2 cortical screws	Compression plate with spring plate	2 cortical screws with washer	SPECT/CT	34	5	Excellent	17/Excellent	N/A	N/A	N/A
12	2 headless screws	Compression plate with spring plate	2 cortical screws	MRI	85	4	Excellent	16/Excellent	N/A	N/A	N/A
13	2 cortical screws	N/A	2 cortical screws with washer	SPECT/CT	38	7	Good	13/Fair	Grade 1	N/A	N/A
14	2 cortical screws	Compression plate with spring plate	2 cortical screws	SPECT/CT	56	8	Excellent	15/Good	Grade 1	N/A	N/A
15	3 cortical screws	Compression plate with spring plate	3 cortical screws with washer	SPECT/CT	72	5	Good	15/Good	Grade 1	N/A	N/A
16	4 cortical screws	Compression plate with spring plate	2 cortical screws with washer	MRI	67	5	Good	14/Good	N/A	Irritation for GT osteotomy site	GT screw removal
17	3 cortical screws	Compression plate	2 cortical screws with washer	SPECT/CT	45	5	Good	16/Good	N/A	N/A	N/A
18	2 headless screws	Compression plate with spring plate	2 cortical screws	SPECT/CT	93	6	Fair	14/Fair	Grade 1	N/A	N/A
19	2 headless screws	Compression plate with spring plate	2 cortical screws	SPECT/CT	90	7	Excellent	15/Good	N/A	N/A	N/A
20	3 cortical screws	Compression plate with spring plate	2 cortical screws with washer	SPECT/CT	51	6	Good	14/Fair	N/A	N/A	N/A
21	2 headless screws	Compression plate with spring plate	2 cortical screws with washer	SPECT/CT	126	7	Good	15/Good	Grade 2	N/A	N/A
22	3 cortical screws	2 cortical screws	3 cortical screws with washer	SPECT/CT	43	10	Fair	13 /Fair	N/A	N/A	N/A
23	2 cortical screws	Compression plate with spring plate	2 cortical screws with washer	MRI	54	8	Good	16/Good	N/A	N/A	N/A
24	2 headless screws	Compression plate with spring plate	3 cortical screws	MRI	40	7	Good	15/Good	N/A	N/A	N/A
25	2 headless screws	Compression plate with spring plate	2 cortical screws	SPECT/CT	59	6	Fair	14/Fair	Grade 2	N/A	N/A
26	2 headless screws	Compression plate	2 cortical screws	SPECT/CT	123	7	Good	13/Fair	N/A	N/A	N/A
27	2 headless screws	Anchor suture fixation	2 cortical screws	SPECT/CT	119	5	Excellent	16/Good	N/A	N/A	N/A
28	1 headless screw	Compression plate	2 cortical screws with washer	SPECT/CT	53	6	Fair	15/Good	N/A	N/A	N/A
29	3 cortical screws	Anchor suture fixation	2 cortical screws with washer	MRI	46	6	Good	15/Good	N/A	N/A	N/A
30	3 cortical screws	Compression plate	2 cortical screws with washer	SPECT/CT	61	4	Good	14/Fair	N/A	Irritation for GT osteotomy site	GT screw removal
31	3 cortical screws	Compression plate with spring plate	2 cortical screws with washer	SPECT/CT	61	8	Good	17/Excellent	N/A	N/A	N/A
32	3 cortical screws	Compression plate with spring plate	3 cortical screws	MRI	53	7	Fair	13/Fair	Grade 2	N/A	N/A
33	4 cortical screws	Compression plate	2 cortical screws with washer	SPECT/CT	52	4	Excellent	17/Excellent	N/A	N/A	N/A
34	3 cortical screws	Compression plate	3 cortical screws with washer	SPECT/CT	35	None	Poor	8/Poor	Grade 3	AVN	THRA

AVN, avascular necrosis of the femoral head; F/u, follow-up; N/A, not applicable; MRI, magnetic resonance imaging; SPECT, single photon emission computed tomography; CT, computed tomography; GT, greater trochanter; THRA, total hip replacement arthroplasty

## Results

All fractures were caused by high-energy trauma: automobile collisions in 32 patients and fall from a height (10 m) in 2 patients. Regarding the Pipkin’s classification, 7 patients had type II fractures, 2 had type III fractures, and 25 had type IV fractures. Posterior wall fractures were accompanied by associated acetabular fractures in Pipkin type IV fractures (Table 1).

The union of acetabular fractures and femoral head fractures was radiologically confirmed by 6.1 months on average (range 4–10 months; Table 2) postoperatively in 32 patients, except in two patients who developed femoral head AVN. Nonunion of the GT fragment was observed in one patient. On radiologic evaluation based on the Matta classification, good or excellent outcomes were achieved in 27 patients. Clinically, the average Merle d'Aubigné–Postel score was 14.4 points (range, 8–17 points; maximum 18 points). According to the Thompson–Epstein Scale, the clinical outcome was good or better in 22 patients, fair in 10, and poor in 2. No additional nerve injury or surgery-related infection was observed. Among the four patients with sciatic nerve injury at the time of fracture, two showed complete recovery, one showed incomplete recovery, and the other one showed no improvement in the final follow-up observation.

Among the 11 (32.4%) patients who developed HO, only one had grade III or higher HO (i.e., affecting the function of the hip joint). During follow-up, femoral head AVN was detected in two patients. Total hip arthroplasty was performed at 14 months on average after the initial surgery in these two patients, both of whom had an associated displaced femoral neck fracture. In three patients, fixation screws caused irritation at the GT osteotomy site. Thus, the screws were removed at 8.7 months on average (range, 8–10 months) after the initial surgery. One patient underwent refixation because of nonunion at the GT osteotomy site. AVN development was investigated using MRI in 8 patients and SPECT/CT in 26 patients, and it was confirmed in 2 of the 34 (5.9%) patients.

**Fig. 2:**
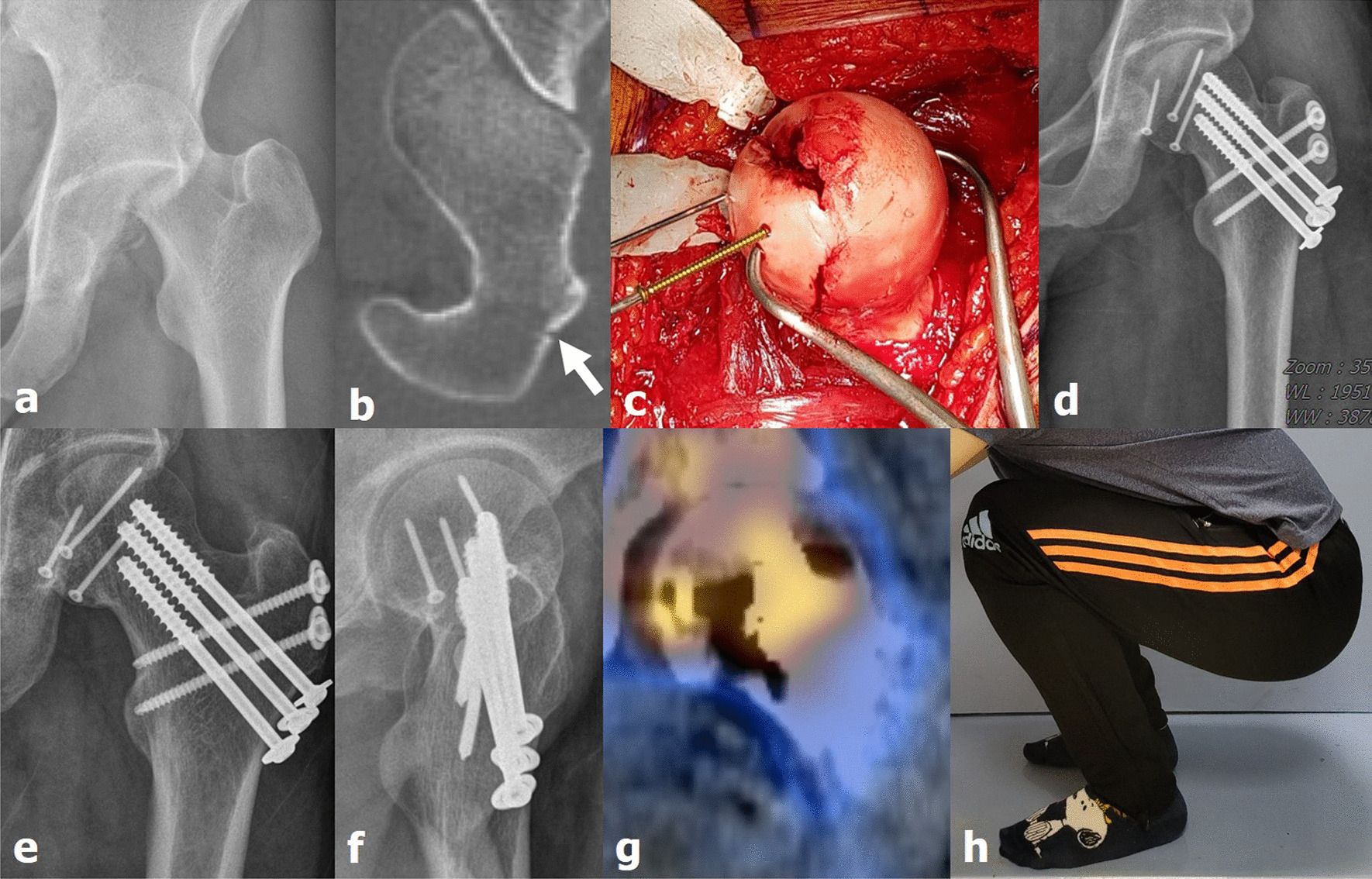
(**a**, **b**) Pipkin type III femoral head fracture and dislocation and femoral neck fracture (white arrow) in the left hip joint owing to an in-car traffic accident (case 9, a 31-year-old man). (**c**) After performing surgical hip dislocation with trochanteric flip osteotomy, the femoral head was exposed and fixed with cortical screws. (**d**) Femoral neck fracture was fixed using three cannulated screws. (**e**, **f**) Bone union was achieved 4 months after surgery, and (**g**, **h**) blood supply to the femoral head was found to be well maintained on single-photon emission computed tomography/computed tomography. The patient fully recovered range of motion

**Fig. 3:**
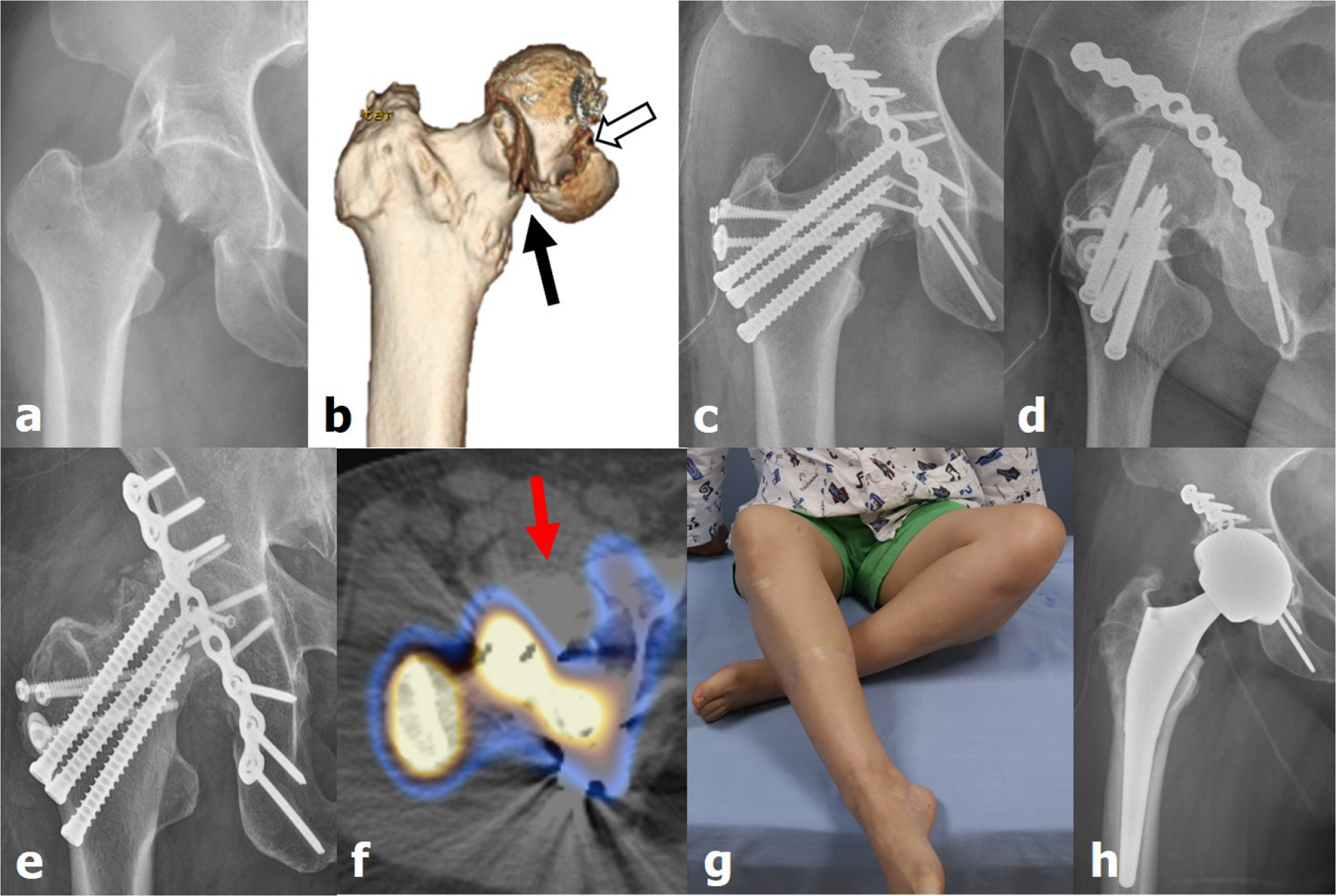
(**a**, **b**) Pipkin type IV femoral head fracture and dislocation associated (white arrow) with a displaced femoral neck fracture (black arrow) of the right hip joint owing to an in-car traffic accident (case 34, a 47-year-old woman). (**c**, **d**) After performing surgical hip dislocation with trochanteric flip osteotomy, the femoral neck and head were fixed and compression plating was performed for the acetabular fracture. (**e**, **f**) Bone union was achieved 8 months after surgery; however, reduced blood flow (red arrow) in the femoral head was observed on single-photon emission computed tomography/computed tomography. (**g**, **h**) The patient complained of limited range of motion and pain in the right hip joint, and eventually underwent total hip arthroplasty

The functional score was the only factor that correlated with all complications, including femoral head AVN and HO (Table 3, *p* = 0.003). Factors, such as age, injury mechanism, presence of associated injury, failure of reduction, time to reduction, and associated acetabular fracture were predicted to be correlated with femoral head AVN during multivariate analysis; however, no statistically significant correlation was found between AVN and these factors. Femoral neck fracture was the only factor that significantly influenced the development of femoral head AVN (Table 4; *p* < 0.001). Three of the patients had femoral neck fractures, of whom two developed femoral head AVN (Figs. 2 and 3). Those two patients had displaced femoral neck fractures (Garden type IV). The factors identified to influence the postoperative functional score were age at the time of injury (*p* = 0.026), associated acetabular fracture (*p* = 0.040), femoral head AVN (*p* = 0.017), and radiologic outcome (*p* = 0.017), with statistically significant correlations (Table 5).

**Table 3:** Risk factor analysis of complications (any, including avascular necrosis and heterotropic ossification) (N = 34)

Variable	Total	No	Yes	*p* value
Sex, n (%)				
Female	6 (100%)	5 (83.3%)	1 (16.7%)	0.237
Male	28 (100%)	16 (57.1%)	12 (42.9%)	
Age (years)	37.91 ± 15.87 (16–77)	39.38 ± 16.38 (16–77)	35.54 ± 15.35 (17–65)	0.478
Injury mechanism				
Traffic accident	32 (100%)	20(62.5%)	12 (37.5%)	0.626
Fall	2 (100%)	1 (50.0%)	1 (50.0%)	
Combined injury				
No	16 (100%)	12 (75.0%)	4 (25.0%)	0.126
Yes	18 (100%)	9 (50.0%)	9 (50.0%)	
Pipkin classification				
II	7 (100%)	5 (71.4%)	2 (28.6%)	0.808
III	2 (100%)	1 (50.0%)	1 (50.0%)	
IV	25 (100%)	15 (60.0%)	10 (40.0%)	
Femoral neck fracture				
No	31 (100%)	20 (64.5%)	11 (35.5%)	0.322
Yes	3 (100%)	1 (33.3%)	2 (66.7%)	
Acetabular fracture				
No	9 (100%)	6 (66.7%)	3 (33.3%)	0.525
Yes	25 (100%)	15 (60.0%)	10 (40.0%)	
Closed reduction				
Failed	6 (100%)	2 (33.3%)	4 (66.7%)	0.136
Within 6 h	17 (100%)	10 (58.8%)	7 (41.2%)	
After 6 h	11 (100%)	9 (81.8%)	2 (18.2%)	
Bone union (months)	6.24 ± 1.52 (4–10)	6.05 ± 1.60 (4–10)	6.54 ± 1.39 (4–9)	0.310
Functional score (Merle d’Aubigne–Postel score)	14.44 ± 1.63 (8–17)	15.19 ± 1.25 (13–17)	13.23 ± 2.24 (8–15)	0.003

**Table 4:** Multivariate regression analyses of cases with avascular necrosis of head

	Beta	F	*p* value
Sex (male)	0.059	0.255	0.617
Age (years)	0.080	0.956	0.337
Injury mechanism (traffic accident)	0.011	0.135	0.716
Combined injury (yes)	− 0.003	0.002	0.968
With neck fracture	0.819	26.014	0.000
With acetabular fracture	0.095	0.865	0.361

**Table 5:** Multivariate regression analyses of functional score (Merle d’Aubigne–Postel score)

	Beta	Standard Error	p-Value	VIF
Age (years)	0.039	0.016	0.026	2.211
Sex (male)	− 0.574	0.667	0.399	2.155
Injury mechanism (fall)	− 0.341	0.921	0.714	1.564
Combined injury (yes)	− 0.185	0.526	0.729	2.299
With neck fracture	0.293	1.172	0.805	3.686
With acetabular fracture	1.146	0.526	0.040	1.797
Complication (AVN)	− 3.817	1.485	0.017	4.067
Complication (HO)	− 0.413	0.489	0.407	1.742
Complication (others)	− 0.960	0.648	0.152	1.453
Time to bone union	− 0.331	0.184	0.086	2.534
Radiologic outcome	0.818	0.317	0.017	2.267

R square: 0.817, Durbin–Watson: 2.045, analysis of variance F-value 8.907 (*p* = 0.000).

VIF, variance inflation factor; AVN, avascular necrosis; HO, heterotopic ossification

## Discussion

FHFD, which is caused by high-energy injury, is very complicated and difficult to treat because it requires prompt treatment, extensive approach to the muscles surrounding the hip joint, and anatomic reduction and rigid fixation due to the nature of intra-articular fractures in addition to the demand for trochanteric flip osteotomy in some patients [3, 21]. Among the 34 patients with FHFD, good outcomes were achieved for SHD with TFO in 32 patients, except 2 patients in whom AVN was caused by the association of the femoral neck fracture, demonstrating that SHD with TFO is a safe and useful approach for the treatment of FHFD.

In FHFD, three major complications are considered to warrant special attention: AVN, post-traumatic osteoarthritis, and HO [22, 23]. Post-traumatic osteoarthritis and HO can be treated conservatively or surgically, depending on the severity; however, in most cases, AVN caused by trauma requires artificial joint surgery, indicating the importance of analyzing the risk factors for AVN in patients with FHFD [24]. Among the fractures classified according to the Pipkin system, type III fractures (i.e., femoral neck fractures with femoral head fractures) have unfavorable clinical and radiologic outcomes [25]. Simultaneous fractures of the femoral neck and head contribute to difficulty in performing reduction and fixation, and AVN can develop owing to medial femoral circumflex artery injury that occurs with the fracture [26]. In this study, femoral neck fractures frequently caused complications with two of the three (66.7%) patients with femoral neck fractures developing AVN.

The anterior approach has the advantage of being convenient for internal fixation because most femoral head fragments are located on the anteromedial side [27, 28]. However, Epstein et al. suggested that the anterior approach can damage the blood flow on the anterior side in addition to the blood flow on the posterior side, which had already been damaged by posterior hip joint dislocation [29]. Moreover, the anterior approach provides limited visualization of the operative field because it does not expose the entire femoral head. It might also provide insufficient fixation because it does not offer sufficient angles for fixation screw insertion when multidirectional screw fixation is required, owing to comminuted femoral head fragments. Giannoudis et al. systematically reviewed AVN rate for the approach in 153 patients with femoral head fracture in 11 studies, and the frequency of AVN development was 3.67 times higher with the posterior approach than with the anterior approach and 2.24 times higher than that with TFO. Additionally, the frequency of post-traumatic arthritis was higher with the anterior and posterior approaches than with TFO by 20.3 times (*p* = 0.04) and 30.6 (*p* = 0.018) times, respectively [9]. This proves that TFO is safe and provides a wide field of view for anatomic reduction and stable fixation.

The correlation between the time to femoral head reduction, which is known to influence functional outcomes, and the Pipkin classification was not statistically significant in the current study; however, a significant correlation was found between functional outcomes and age, associated acetabular fracture, AVN development, and radiologic results [4, 30]. This may be because older age is associated with more difficult postoperative rehabilitation, consequently, a longer time to return to daily activities. Moreover, the presence of an associated acetabular fracture with FHFD indicates serious hip joint damage due to high-energy injuries [31]. Marchetti et al. found no significant difference in a comparative analysis of closed reduction within and after 6 h [32]. This means that blood flow disturbance caused by femoral neck fractures has a large influence on the outcome [6].

In AVN diagnosis, SPECT/CT and MRI are useful methods; however, they have some limitations. MRI is currently the most accurate method for AVN diagnosis [33]. Compared with simple radiography, MRI allows a much earlier diagnosis of necrosis, specifies the location and size of the lesions, which are crucial for a more accurate determination of prognosis or treatment, and aids in the differential diagnosis of subchondral stress fractures or transient osteoporosis of the hip joint (bone marrow edema syndrome); this may present a similar pattern to AVN. However, MRI is relatively expensive, and signal blurring caused by the metals used to fix the fracture site of the femoral head and acetabulum may interfere with accurate diagnosis [34]. SPECT has higher accuracy than simple bone scanning; however, it does not specify the size or location of necrosis. To overcome this limitation, the SPECT/CT examination method, which combines SPECT and CT, was developed. This method evaluates the blood flow status in the bone while precisely localizing the necrotic site using CT, and it has a lower examination cost (by approximately one-third) than MRI. Additionally, the interference of implants is less than that present in MRI, thereby allowing for accurate examination even after implant insertion [35]. Park et al. reported that SPECT/CT has a diagnostic value in predicting the occurrence of AVN after femoral neck fractures [18]. When Ganz introduced the method for TFO, he devised it to solve the impingement that occurred in the femoral head or acetabulum, and AVN was confirmed by simple radiography [36]. In this study, MRI or SPECT/CT was performed for the first time to accurately identify the lesion site for FHFD.

This study has several limitations. First, this was a retrospective study in which data were obtained from the patients’ medical records, and only patients with FHFD who underwent SHD were included. The incidence of AVN varied according to the patient’s age, sex, and severity of damage [32]. In this study, there was a large difference in the number of male and female patients and a large age span among the study participants. Hence, a selective bias may have been introduced during data collection. Second, the incidence of FHFD (i.e., number of patients with FHFD treated at our hospital) was relatively low, no control group was included, and patient compliance was not considered. Third, the relatively short follow-up period may have limited our evaluation of the clinical and radiological outcomes in patients who were followed up for > 2.5 years. Nonetheless, we believe that our findings from an average follow-up of 5 years provide sufficient evidence for the effectiveness of SHD as a surgical technique, in view of Brav et al.’s report that 98% of AVN developed within 1 year in patients with traumatic hip dislocation [37].

## Conclusions

TFO with SHD is a safe approach with a minimal risk of damage to the blood supply of the femoral head. It may also provide a wide operative field by visualizing the entire region of the femoral head, resulting in anatomical reduction. However, the association between displaced neck fractures still requires careful decision making related to joint replacement.

## Data Availability

The datasets generated and/or analyzed during the current study are not publicly available because of restricted access to our hospital database but are available from the corresponding author upon reasonable request.

## Abbreviations

AVN: Avascular necrosis
HO: Heterotopic ossification
SHD: Surgical hip dislocation
TFO: Trochanteric flip osteotomy
FHFD: Femoral head fracture and dislocation
SPECT/CT: Single-photon emission computed tomography/computed tomography
MRI: Magnetic resonance imaging
GT: Greater trochanter
